# AMOC decline and recovery in a warmer climate

**DOI:** 10.1038/s41598-023-43143-5

**Published:** 2023-09-23

**Authors:** Paulo Nobre, Sandro F. Veiga, Emanuel Giarolla, André L. Marquez, Manoel B. da Silva, Vinícius B. Capistrano, Marta Malagutti, Julio P. R. Fernandez, Helena C. Soares, Marcus J. Bottino, Paulo Y. Kubota, Silvio N. Figueroa, José P. Bonatti, Gilvan Sampaio, Fernanda Casagrande, Mabel C. Costa, Carlos A. Nobre

**Affiliations:** 1 Center for Weather Forecasting and Climate Studies (CPTEC), National Institute for Space Research (INPE), Cachoeira Paulista, São Paulo 12630-000 Brazil; 2 School of Atmospheric Sciences and Key Laboratory of Mesoscale Severe Weather/Ministry of Education, Nanjing University, Nanjing, China; 3 Institute of Physics, Federal University of Mato Grosso do Sul (UFMS), Campo Grande, Mato Grosso do Sul 79070-900 Brazil; 4 Institute for Advanced Studies, University of São Paulo, São Paulo, São Paulo 05508-050 Brazil

**Keywords:** Climate and Earth system modelling, Physical oceanography, Projection and prediction

## Abstract

This study presents novel insight into the mechanisms of Atlantic Meridional Overturning Circulation (AMOC) reduction and its recovery under a warmer climate scenario. An one-thousand-year-long numerical simulation of a global coupled ocean–ice–atmosphere climate model, subjected to a stationary atmospheric radiative forcing, depict a coherent picture of the Arctic sea ice melting as a trigger for the initial AMOC reduction, along with decreases in the northward fluxes of salt and heat. Further atmospheric-driven ocean processes contribute to an erosion of the stable stratification of the fresher, yet colder waters in the surface layers of the North Atlantic, contributing to the recovery of a permanently altered AMOC.

## Introduction

The scientific scrutiny of the Atlantic Meridional Overturning Circulation (AMOC) as a driver of world climatic stability and change has sharply increased recently^1–7^. Several studies based on both paleoclimatic data and model simulations concur in proposing mechanisms for AMOC reduction^8–11^ or eventual collapse^12–17^ in a warmer climate. According to the concept of the AMOC being driven by density gradients associated with deep-water formation in the North Atlantic (NATL)^18, 19^, an eventual weakening of the AMOC can be seen as a direct result of the reduction of surface water density due to warming or salinity decreases of the upper ocean layers^1^. Yet, the AMOC recovery has been suggested by several studies. One among them, for example, attributes the recovery to a northward transport of anomalous warmer water at depth, into the NATL region, that results in a destabilization of ocean stratification, restarting then the convection^1^. Other study, on the other hand, suggest that the recovery is driven by ocean-only processes based on downward advection and mixing of freshwater by the still-active AMOC, that lead to an erosion of the stratification in the NATL, which generates convection^8^.

**Figure 1:**
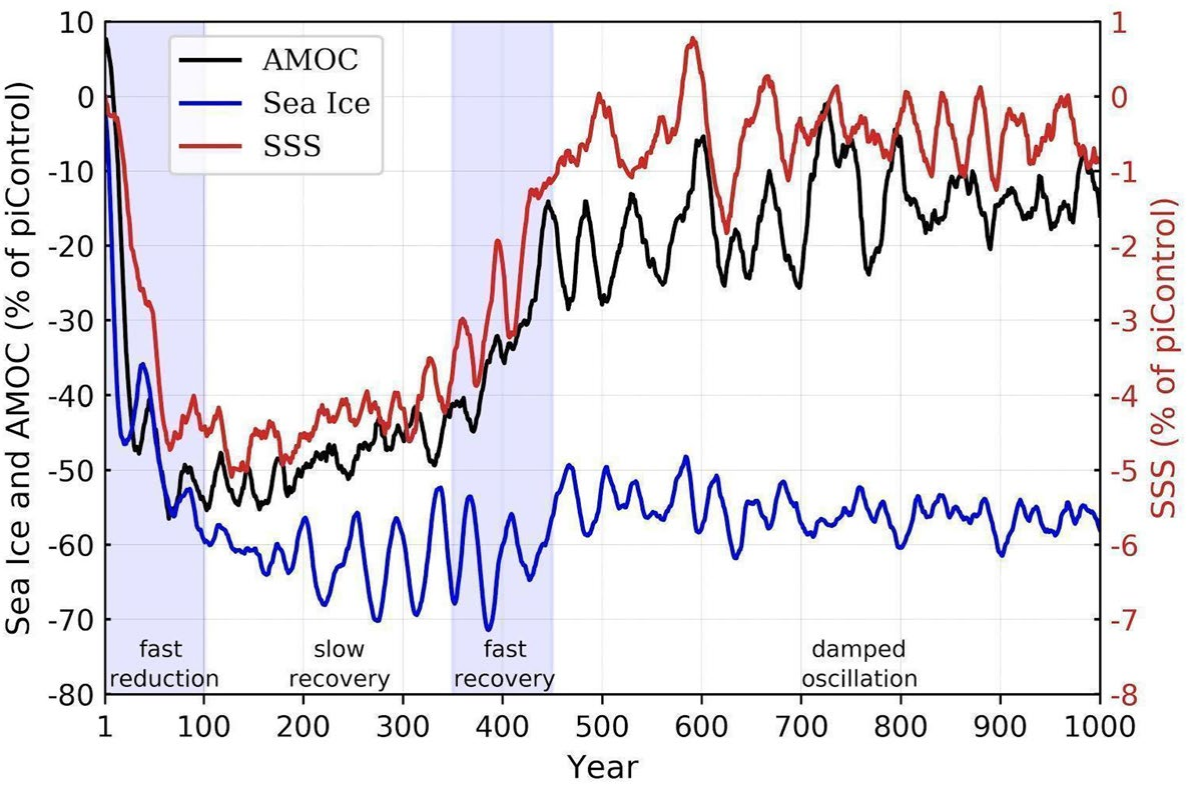
Abrupt4xCO_2_ minus piControl percent change-time series relative to the piControl run of the Arctic sea-ice volume in March (blue), the AMOC strength (black), and the NATL (60° W–20° W, 40° N–60° N) SSS (red) lines are plotted. The named periods, i.e., fast reduction, slow recovery, fast recovery, and damped oscillation, are indicated at the base of the figure.

Here we propose that the AMOC demise is caused by a Summer-Fall pulse of warm air/water temperature, followed by a Winter sea ice melting over the Arctic and a Spring southward advection of fresher/cooler waters by ocean currents. The recovery of the AMOC, on the other hand, is attributed to the vertical stratification erosion caused by the wind driven Ekman pumping, bringing denser (saltier) waters to the surface, by the AMOC itself, which is much weaker but not totally collapsed, and by the solar radiation warming of surface waters, slowly dissipating the cold surface anomaly. Those statements are based on a set of one-thousand-year-long numerical simulation of the Brazilian Earth System Model (BESM2.5)^20–23^, with two stationary atmospheric forcings, the pre-industrial (piControl) and the abrupt four times atmospheric CO_2_ concentration increase (Abrupt4xCO_2_) of the CMIP5 protocol^24^. The Abrupt4xCO_2_ stationary atmospheric forcing experimental design enables climate considerations of transient processes that act in the global climate system. The model, which demonstrated a bistable equilibria characteristic^2, 25^ (Supplementary Fig. 1), underwent four phases; fast reduction, slow recovery, fast recovery, and damped oscillations, depicted in Fig. 1 and discussed below.

**Figure 2:**
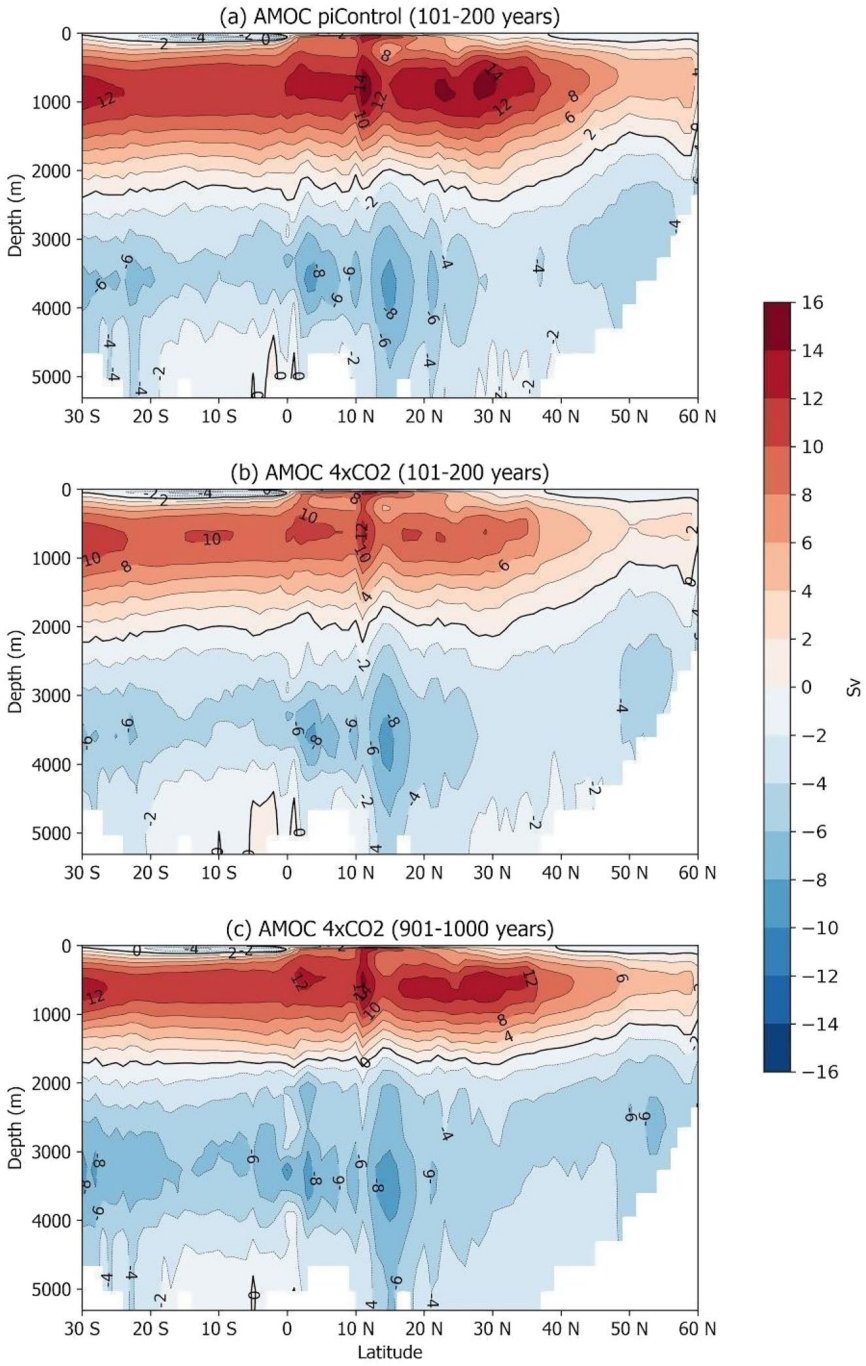
The AMOC meridional-vertical profiles (**a**) for the piControl simulation averaged over the period from 1 to 100 years and for the Abrupt4xCO_2_ simulation averaged over the periods (**b**) 101–200 and (**c**) 901–1000 years.

The AMOC meridional mean profiles for control and perturbed conditions, the latter in the first and last 100 years of simulation, are shown in Fig. 2.

## Results and discussion

### AMOC weakening mechanisms

Links between Arctic sea-ice loss and AMOC reduction have been pointed out by several studies. Consistent with the findings of previous studies^3, 26–31^, the Abrupt4xCO_2_ BESM2.5 simulation predicts a time-evolving AMOC structure related to a concurrent decrease in Arctic sea ice volume and a reduced AMOC (Supplementary Fig. 2), which are components of a transient response (Fig. 1) to stationary atmospheric CO_2_ perturbation. Several CMIP5 and CMIP6 models incorporating Abrupt4xCO_2_ forcing show similar AMOC reduction rates^32^ (Supplementary Fig. 2b). These effects are signals of climate change, as indicated by BESM2.5 and other CMIP5/6 model simulations, as well as by recent observational data and studies^5^.

When the atmospheric CO_2_ concentration is abruptly quadrupled in BESM2.5 numerical simulation, the global ocean surface initially warms quickly and then asymptotically approaches a new equilibrium temperature, approximately 4 °C warmer than that of the piControl (Supplementary Fig. 3a). The global mean surface salinity decreases steadily in the Abrupt4xCO_2_ experiment during the one-thousand-year period (Supplementary Fig. 3b). We speculate that the surface freshening shown in the several areas depicted in Supplementary Fig. 7e–h is in part a consequence of an enhanced upper ocean convection (i.e., saltier/denser water parcels sink and fresher/lighter parcels upwell) as the result of sea surface temperature (SST)-induced augmented evaporation. While the global mean surface salinity steadily freshens, eventually reaching an equilibrium in the Abrupt4xCO_2_ scenario, at Cape Farewell in the NATL (an important region for water mass formation)^33^ abrupt cooling/freshening followed by a sudden return and overshoot of the piControl values also occurs (Supplementary Fig. 3c,d). Such fast local changes over the NATL suggest that advective oceanic processes are at play as a response to the steady, globally uniform radiative forcing imposed in the model atmosphere.

The initial weakening (and later strengthening) of the AMOC is concurrent with changes in the NATL surface density^34^.

The AMOC weakens in the first decades of the simulation as the NATL surface waters become fresher (i.e., lighter) after approximately 20 years of simulation (Fig. 1), with the reduction in density due primarily to salinity depletion rather than to temperature changes^1^, an aspect that may be model dependent^35, 36^. In fact, estimations of the contribution due to changes in temperature and salinity separately (figure not shown) demonstrated more clearly that the salinity change is the dominant factor for the total density change in that region. Based on our simulations, we suggest that the sequential effects of two main mechanisms explain the initial halving of the AMOC strength: (1) southward advection of Arctic sea ice melt water, at a rate of ~ 0.1 Sv (figure not shown) during the first 150 years after CO_2_ quadrupling, reduces NATL salinity/density; (2) upon the establishment of a fresher surface water pool, the climatological density front at 40° N is displaced southward (Supplementary Fig. 4a), the veering of the Gulf Stream (GS) at lower latitudes in the Abrupt4xCO_2_ scenario is seen as an additional driver of the reductions in the net upper ocean meridional heat and salt transport from the lower latitudes into the NATL^27^.

**Figure 3:**
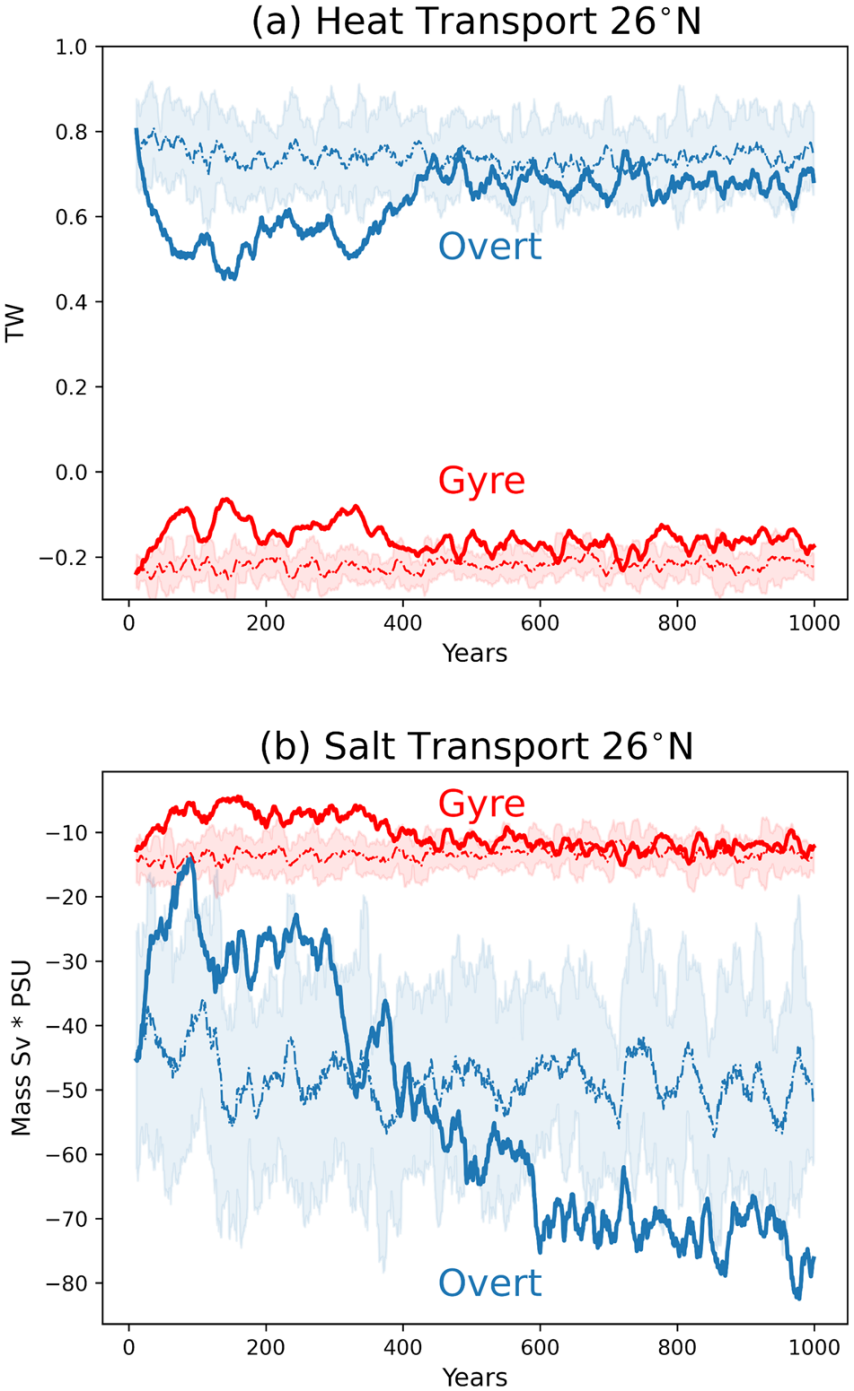
Meridional ocean (**a**) heat and (**b**) salt transport in the North Atlantic cross-section (100° W–15° E), along latitude 26° N, indicating the reduction and recovery of both heat and salt transport of the Abrupt4xCO_2_ (solid lines) in relation to the piControl simulation (dashed lines). The transports were decomposed into “overturning” (blue lines) and “gyre ” (red lines) components, according to reference^37^. All time series have been smoothed over a 10-year running mean. The shades represent lower and higher values of the piControl standard deviation multiplied by two.

Using the method proposed by reference^37^, the total heat transport is decomposed into four components; overturning, gyre effect, seasonal overturning, and transient eddies. Here we consider the first two at 26° N (Fig. 3). The weakening of the heat transport for both components during the initial 400 model simulation years is evident (Fig. 3a), with the reduction for the overturning component approximately 4 times larger than the gyre. Applying the same method for the salinity transport, a reduction of the southward salt transport across 26° N for both components is also noted (Fig. 3b). However, while the gyre salt transport approaches the control run values after the initial 400 simulation years, the overturning component presents a steep increase of southward salt transport after the initial relaxation, overshooting the control run transport.

**Figure 4:**
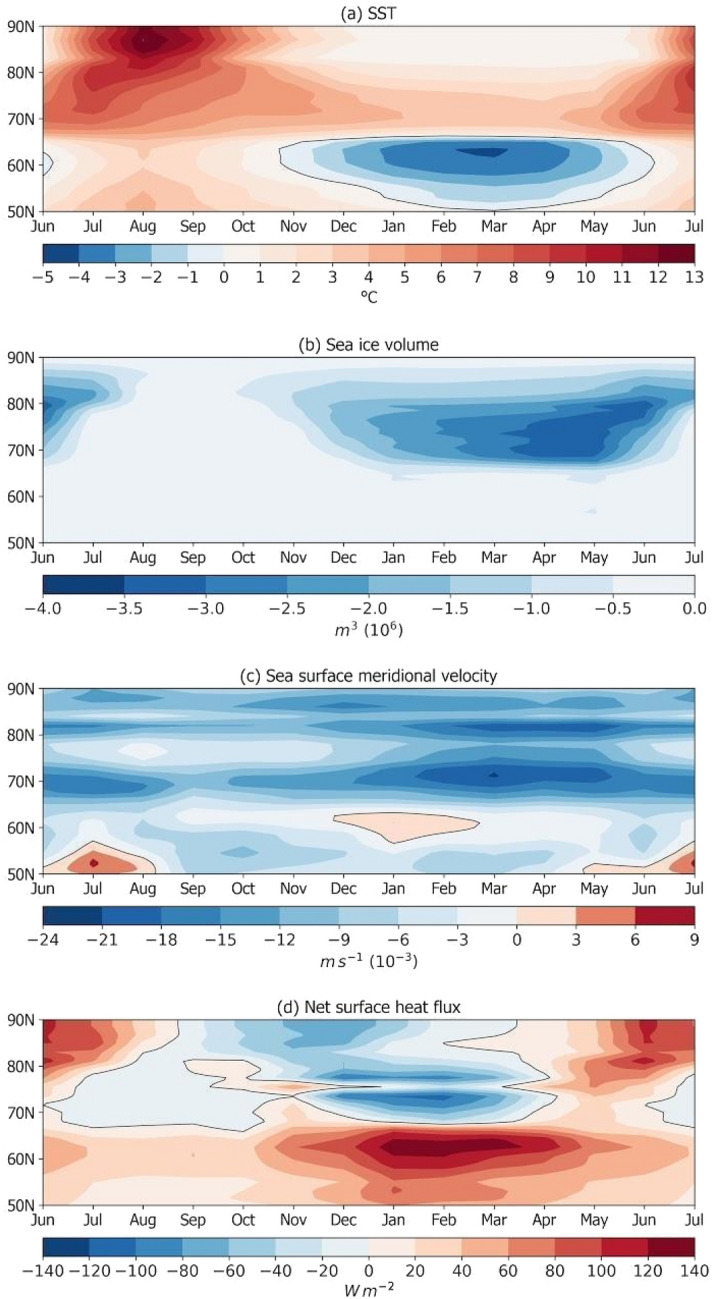
Latitude-time diagrams showing the Abrupt4xCO_2_-piControl monthly climatologies of (**a**) the SST (°C), (**b**) sea-ice volume (10^6^ m^3^), (**c**) meridional surface ocean velocity (m s^−1^), and (**d**) net surface heat fluxes (W m^−2^). The monthly climatologies were computed for the simulation years 121 and 150.

Turning to the mechanics of the NATL cold anomaly, the SST diagram (Fig. 4a) shows that a heat pulse precedes the presence of cold waters in the North Atlantic, which is attributed to a heat pulse in the air temperature due to the quadrupling CO_2_ scenario (figure not shown). After then, there is an extended period of sea ice melting (during spring, Fig. 4b) and followed by enhanced southward ocean currents (Fig. 4c) which advect the cold waters over the NADW formation regions at the Cape Farewell. Furthermore, the NATL SST anomalies drive heat flux from the atmosphere to the ocean, heating the ocean surface over the area of cooler SSTs^34, 38^ (Fig. 4d). This sequence of events suggests that coupled ocean–ice–atmosphere processes take place, resulting in the spring–summer pool of cold anomalies over the NATL following the winter warming in the Abrupt4xCO_2_ experiment. These processes impact the AMOC until the yearly reduction in sea-ice volume reaches an interannual winter minimum value and the surplus of melt waters into the NATL reaches zero, mostly because the sea-ice volume that melts in one summer refreezes in the subsequent winter, helping to end the AMOC weakening phase.

### AMOC recovery processes

After the initial sudden cooling and freshening of the NATL surface waters, an initially gradual, and then fast recovery of the sea surface salinity (SSS), SST, and AMOC towards the piControl conditions took place, but for the Arctic sea ice (Supplementary Fig. 5).

**Figure 5:**
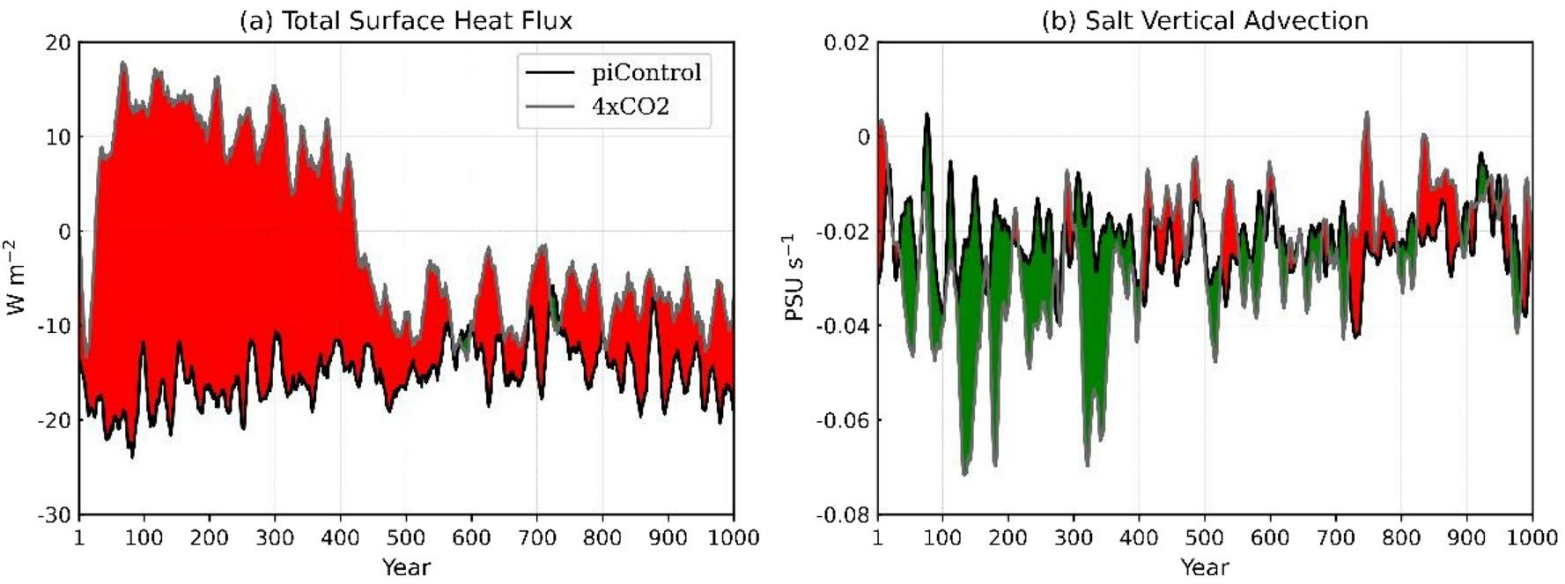
Time series of (**a**) the total surface heat transfer between the atmosphere and the surface of the ocean (the sum of net shortwave, longwave, sensible, and latent heating) over the NATL and (**b**) salt vertical advection ($-W\times \frac{dS}{dz}$) within the upper 200 m of the ocean averaged over the area from 60° W–20° W, 40° N–60° N. The piControl (black lines) and Abrupt4xCO_2_ (gray lines) time series have been smoothed over 10-year running mean. The green and red shades represent Abrupt4xCO_2_ values lower and higher than the piControl values, respectively.

With respect to temperature, the emerging imbalance in the latent and sensible heat fluxes over the cold pool of the NATL warms the upper ocean in the Abrupt4xCO_2_ scenario. Such NATL warming occurs as a result of an excess of about 30 W m^−2^ on average over the NATL relative to the conditions in the piControl that lasts for the first 400 years of model integration (Fig. 5a). With respect to salinity, enhanced convection in the uppermost 200 m of the ocean surface layers (green shades in Fig. 5b) erodes the horizontal, buoyant fresher surface ocean, mixing the fresher surface water with the saltier water in the deeper layers.

For the fast recovery period indicated in Fig. 1 (simulation period from 300 to 400 years), the anomalous veering of the GS at lower latitudes observed in the Abrupt4xCO_2_ experiment gradually decreases so the heat transport at 26° N of the gyre component tends to recover (Fig. 3a), but not reaching the initial magnitudes, before the AMOC weakening process. In fact, Fig. 3 indicates that both overturning and gyre components, of the heat and salt transport at 26° N, do not fully return to their initial values. The negative (towards the tropical region) mean salt transport at that location becomes stronger after 400 years of simulation, in the overturning component, which is attributed to changes in the evaporation processes, as discussed before, and in the hydrological cycle^39^.

**Figure 6:**
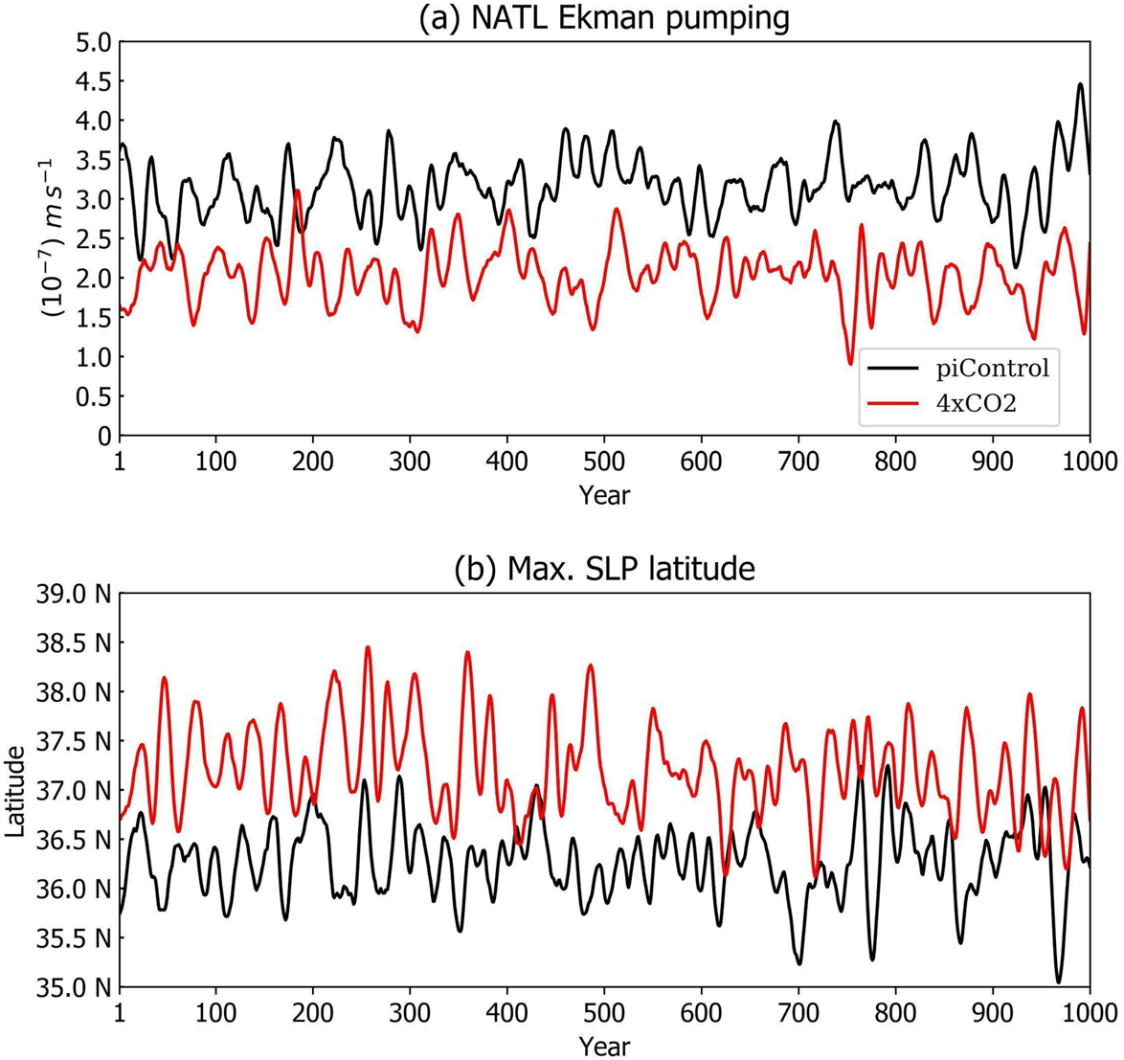
(**a**) Ep (10^−7^ m s^−1^) at the bottom of the oceanic Ekman layer averaged over the NATL (40º N–60º N, 60º W–0º E) and (**b**) the latitudinal position of the north tropical Atlantic high pressure center, 10-year running mean time series. Abrupt4xCO_2_ (red) and piControl (black) lines.

A fast return of the AMOC has been proposed in recent studies^1, 8, 26^; however, some of the physical processes evoked in these studies^1, 8^ differ from other mechanisms presented here. In our analysis, the AMOC recovery is driven by a combination of ocean–atmosphere processes that erode the vertical stratification in the NATL. First, since the AMOC does not completely collapse and is still active, fresh waters from melting ice at high latitudes are transported to deeper regions (there is still oceanic convection). This causes an increase in the density gradient between the denser surface waters advected by the surface AMOC flow, at mid-latitudes, and the less dense deep waters. After a certain time scale (typically some decades), this gradient generates the conditions for a reinvigoration of the AMOC^8^. Therefore, the ocean also plays a role along with the atmosphere feedback in the AMOC recovery. Other processes include wind-driven upper ocean convection, allowing an influx of salty tropical waters into the NATL. In the piControl experiment, the average curl of the surface winds over the border of the Arctic Circle area is positive, indicating upper ocean convergence and downwelling via Ekman pumping (Ep), whilst in the Abrupt4xCO_2_ experiment, the Ep is one-third lower than that of the piControl (Fig. 6a), thus suggesting a reduction of atmospheric-induced downwelling over the region. The reduced curl of the surface winds over the NATL under the Abrupt4xCO_2_ conditions is seen as a result of a systematic northward displacement of the North Atlantic subtropical high-pressure system (Fig. 6b) and the consequential reduction in the mean wind speed (figure not shown). This northward displacement of the North Atlantic subtropical high pressure system is consistent with several observational and modelling studies reporting the widening of the Hadley circulation in a warmer climate^40–42^. Such a reduction in Ep-induced downwelling favors the occurrence of surface layer turbulence^27^ that bring saltier/heavier waters upward (Fig. 5b), thus contributing to faster erosion of the density stratification due to the freshening of the NATL surface layers induced by earlier processes. Based on sensitivity experiments of the effect of wind stress forcing on AMOC, previous studies^43, 44^ observed that AMOC strength reduces when wind stress forcing is reduced and increases when wind stress forcing is increased. In our case, under an already diminished AMOC strength, the reduced Ekman pumping contributes to oceanic effect of restoring the AMOC, however not to the piControl level since the atmospheric conditions (northward displacement of the North Atlantic subtropical high-pressure system) remains through the entire Abrupt4xCO_2_ experiment (Fig. 6).

The numerical evidence for the proposed enhanced convection in the upper ocean layers in the Abrupt4xCO_2_ experiment is also clear in Supplementary Fig. 6, which shows a thicker layer of upward motion at the surface of the NATL and a reduced downwelling in the upper 2000 m during the first 400 years of the model run, coincident with the reduced AMOC strength noted during the same period, compared with the effect seen in the piControl run. The deep convection, in both experiments, is located mainly between latitudes 50°–60° N, and between longitudes 60°–50° W.

### Long-term repercussions in Atlantic structure

During the last six-hundred years of the simulation, the Abrupt4xCO_2_ AMOC not only undergoes decadal scale oscillations (along with the SSS, shown in Fig. 1), but it also exhibits a lasting vertical structural change reflected by an approximately 1000 m shallower northward flowing upper branch, compared with the results of the piControl run (Fig. 2). This shallower upper branch of the AMOC is consistent with the shallower depth of the zero vertical velocity averaged over the NATL (shown in Supplementary Fig. 6).

**Figure 7:**
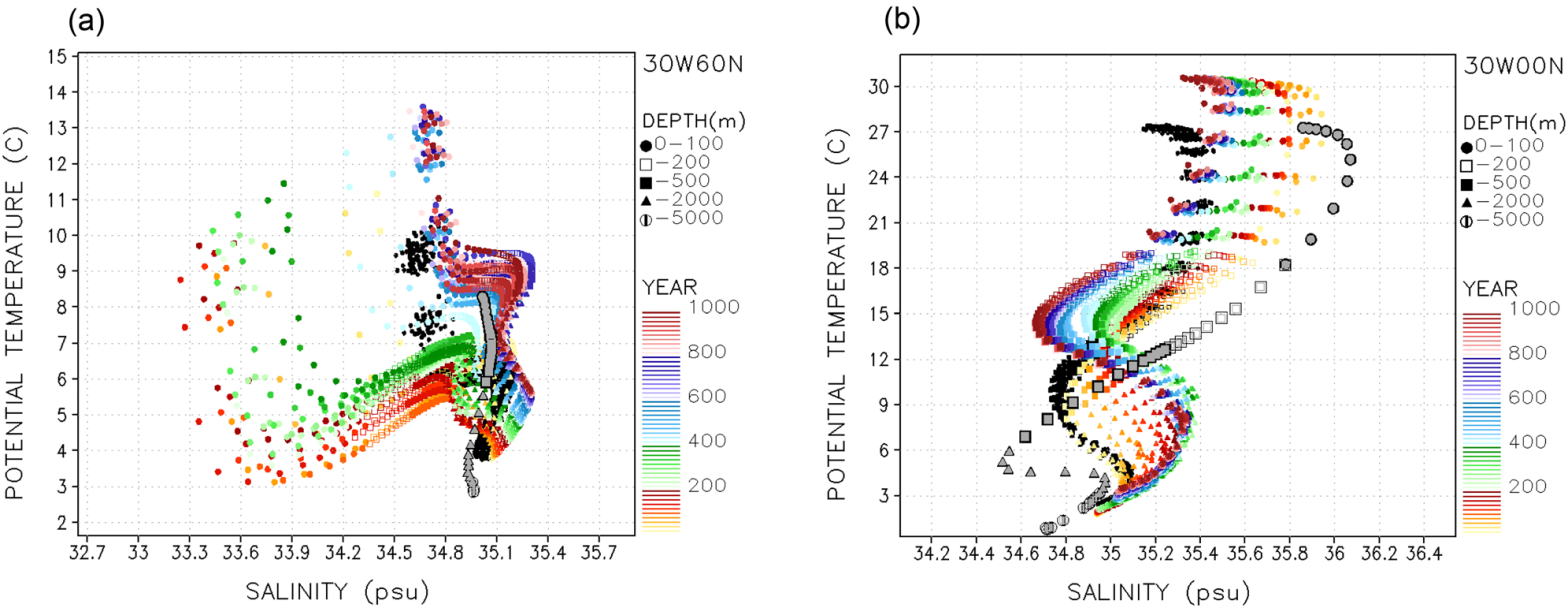
T–S scatter plots at (**a**) 30° W, 60° N and (**b**) 30° W, 0° N for the piControl (black) and Abrupt4xCO_2_ (colors). Depth profiles (depth scale symbols at right) are 10° × 10° lon-lat area averaged and 10-year averages (time color scale at right). The World Ocean Atlas observations annual mean profiles are plotted in gray symbols.

A further indication of the interhemispheric linking role of the AMOC on salinity and temperature variations over the whole Atlantic is shown in the vertical structure T–S diagrams over both the North and Equatorial Atlantic (Fig. 7). The initially marked freshening-cooling of the upper ocean over the NATL shifts rapidly back to the piControl salinity vertical profile around year 400 (Fig. 7a). In this figure, changes in the entire water column temperature and salinity are notable, reflecting the strength of the vertical motions over this area. There is a reverse salinity tendency in the Equatorial (Fig. 7b) and Southern Tropical Atlantic (Supplementary Fig. 7) relative to that shown in the NATL diagram. Also shown in Fig. 7 are the World Ocean Atlas long-term mean T–S values for each depth, as a reference for the BESM2.5 piControl profiles. Other analyzed ocean sites shown in Supplementary Fig. 7 depict consistent warming and freshening trends, coincident with the global atmospheric forcing of a warmer atmosphere.

One notable feature revealed by the time-lapse T–S diagrams shown in Supplementary Fig. 7 is a delay in the manifestations of the NATL deep water (NADW) and the Antarctic intermediate water (AAIW) signatures in the Equatorial Atlantic. While a saltier NADW manifests itself already during the first 200 years of the Abrupt4xCO_2_ run, the freshening of the AAIW occurs only during the second half of the experiment (after year 500). It is noteworthy that the freshening followed by the salting of the NATL is concurrent with the salting followed by the freshening of the tropical Atlantic, whilst the remaining ocean basins show a steady freshening during the one-thousand-year period in this simulation. These effects suggest that AMOC variations might act not only as an interhemispheric modulating mechanism, but that they could also contribute to a modification of the typical water mass T–S signatures in a warmer planet.

## Concluding remarks

Among the main findings of this study is the numerical evidence that AMOC demise and recovery under a warmer climate are consequences of a chain of atmospheric-driven ice-ocean phenomena, explaining, in part, the transient response of the AMOC to the imposed steady and uniform radiative heating resulting from quadrupling the atmospheric CO_2_ concentration. The proposed chain of events are as follows: The warmer 4xCO_2_ atmosphere imposes a reduction in the Arctic sea ice volume. The melted Arctic ice waters freshen and cool the surface North Atlantic waters, limiting the northward inflow of salty waters into higher latitudes. As a consequence, the lighter North Atlantic surface waters reduce North Atlantic Deep Water formation, reducing AMOC strength. With time, the North Atlantic cold pool is eroded by atmospheric radiative heating, while the fresher North Atlantic surface waters are mixed with saltier deeper waters from below by wind-driven Ekman pumping. As the anomalous meridional density gradient decreases, northward flowing salty/warm waters reach the NADW formation regions, reigniting AMOC strength. Besides, since AMOC is not totally collapsed, it contributes to erode the vertical stratification and reinforce itself.

However, uncertainties are normally associated with the use of any single global coupled ocean–ice–atmosphere modeling study. By contrast, this study provides compelling quantitative data explaining the initial AMOC demise that are supported by several other coupled ocean–ice–atmosphere models. These findings suggest a central role for Arctic sea ice melting as a *trigger* and the overturning circulation as a *modulator* of abrupt changes in AMOC strength, both of which are driven by atmospheric processes. Additionally, the concurrent freshening and subsequent-salting of the North Atlantic and the opposing salting and subsequent freshening of the Tropical and South Atlantic are seen as consequences of the AMOC strength variations, interlinking the entire Atlantic meridionally.

Despite the appealing sequence of events described here, the timeframe in which they unfold is in part a consequence of the constraints and characteristics of the global-coupled model used. Other processes not considered here, for example the melting of continental glaciers over Greenland and northern land masses, might greatly shorten the time frame in which the AMOC can present substantial variations. For instance, the current observations of persistent cold anomalies over the North Atlantic, concurrent with warmer than normal northern oceans might indicate that the processes highlighted in this modeling study are already acting upon the Earth's climate.

## Methods

### Coupled ocean atmosphere land ice model

The Brazilian Earth System coupled ocean–atmosphere–land–ice Model version 2.5 (BESM2.5) was used for this research. It comprises component models from INPE/CPTEC (atmospheric and land global models) and NOAA/GFDL (ocean and sea ice global circulation models). The ocean model is the Modular Ocean Model MOM version 4p1 (MOM4p1)^45^, which includes the Sea Ice Simulator (SIS)^46^. The vertical ocean grid has higher vertical resolution in the upper 220 m (with approximately 10 m between the levels), while it gradually increases to approximately 370 m at the deeper layers. The horizontal grid has ¼-degree latitudinal intervals between 10° S and 10° N, increasing uniformly to about 1 degree at 45° and to 2 degrees at 90° of latitude in both hemispheres. The longitudinal grid resolution is 1 degree. The horizontal grid also has a convergence of the grid elements into two poles in latitudes higher than 65° N, a technique referred to as a “tripolar grid”, to avoid the singularity that occurs due to the lack of continents in the northernmost grid elements (i.e., the North Pole). The ocean model settings were based on the coordinated ocean-ice reference experiment (CORE) settings with some modifications as described in^20^. The ocean model spin-up process was a combination of an ocean-solo run of about 70 years with atmospheric climatological and NCEP/NCAR reanalysis forcing fields based on the reference^47^ dataset, plus a subsequent coupled ocean–atmosphere run of about 100 years with similar settings^20, 48, 49^.

The atmospheric component model is the Brazilian Atmospheric Model (BAM)^50^, but with simpler physical parameterizations^22^. The horizontal grid resolution was truncated at triangular wavenumber 62 (approximately 1.875 × 1.875 degrees of resolution at the Equator) and 28 sigma levels unevenly spaced in the vertical dimension. The model performance with the CMIP5 historical experiment settings reproduced the main observed global atmospheric and oceanic variability patterns^21^. The land model is SSiB^51^, which computes the heat and water balances over the continental areas, while the land ice sheet is absent in this version of the model.

Two sets of experiments were performed following the CMIP5 protocol: the piControl (pre-industrial control) and the Abrupt4xCO_2_ (abrupt quadrupling the atmospheric CO_2_ concentration) scenarios, described in^24^. Both experiments simulated approximately 1000 years, with the AbruptCO_2_ starting at (and using the initial conditions of) year 147 in the piControl experiment. Both the monthly and annual means of the output fields were used for the analysis.

### AMOC estimation

The AMOC was obtained using the zonally and vertically integrated volume transport at each depth, given by the following equation:

$$\Psi \left(y,z\right)={\int }_{z}^{\eta }{\int }_{{x}_{w}}^{{x}_{e}}vdxdz\left({\mathrm{m}}^{3}{\mathrm{s}}^{-1}\right)$$

in which *v* is the meridional velocity, *z* is the vertical coordinate increasing upward (from the bottom to the surface), *η* is the height of the free surface, and *x*_*w*_(*z*) and *x*_*e*_(*z*) are the westward and eastward positions of the bathymetry at a certain depth^52^. Since we are assessing the AMOC strength in a set of different models, the maximum strength of the AMOC was obtained as an average value in the region defined by a box delimited by latitudes 25° N–30° N and a depth 600–1000 m.

### Comments on the BESM2.5 AMOC stability

The AMOC stability criterion is defined as the sign of the AMOC freshwater transport at the southern boundary of the Atlantic Ocean^2, 25^

$${F}_{OT}=- \frac{1}{{S}_{0}}\int \overline{\upsilon }<S>dz$$

where $\overline{\upsilon }$ is the zonally integrated baroclinic meridional velocity, and < *S* > and *S*_*0*_ are the zonally averaged and reference salinity, respectively. In this criterion, a negative F_OT_ implies a bistable AMOC^2^. Supplementary Fig. 1 indicates that BESM2.5 has a “bistable AMOC'' characteristic according to this criterion.

### Heat meridional transport

The meridional depth-integrated heat transport was computed via the equation:

$${F}_{Q}=\rho {c}_{P} {\int }_{{L}_{e}}^{{L}_{w}}{\int }_{{H}_{L}}^{{H}_{0}}v\,\theta\,dz\,dx\,\left(W\right)$$

where ${c}_{P}$ is the specific heat capacity, $\rho$ is the sea water density, *L*_*w*_ and *L*_*e*_ are the westward and eastward coordinate positions over which the transport is integrated, ${H}_{0}$ and ${H}_{L}$ are the depths over which the transport is integrated, $v\left(x,z,t\right)$ is the sea water meridional velocity component and $\theta \left(x,z,t\right)$ is the sea potential temperature^52^.

### Salt meridional transport

This factor was computed via the equation:

$${F}_{S}=\rho {\int }_{{L}_{e}}^{{L}_{w}}{\int }_{{H}_{L}}^{{H}_{0}}v\,S\,dz\,dx\,\left(Kg{s}^{-1}\right)$$

where $\rho$ is the sea water density, *L*_*w*_ and *L*_*e*_ are the westward and eastward coordinates positions over which the transport is integrated, ${H}_{0}$ and ${H}_{L}$ are the depths over which the transport is integrated, $v\left(x,z,t\right)$ is the sea water meridional velocity component and $S\left(x,z,t\right)$ is the oceanic salinity^53^.

### Surface of the ocean heat transfer

The surface energy budget between the atmosphere and the ocean was computed by the net (downward minus upward) shortwave and longwave radiative fluxes plus the latent and sensible heat fluxes. Positive values indicate fluxes from the atmosphere into the ocean.

### Vertical mixing of salt

This factor was defined as the vertical integration of the vertical salt advection, $-w\frac{dS}{dz}$, as

$${\int }_{z=200m}^{z=0m}-w\frac{dS}{dz}$$

averaged over the area delimited by 40° N–60° N and 60° W–20° W.

### Ekman pumping

The Ekman pumping was defined as

$${W}_{E}=\frac{1}{(\rho f)(\nabla \times \tau )}$$

where $\rho$ is considered here as the upper layer density, $f$ is the Coriolis parameter and $\tau$ is the wind stress vector.

### Time series of the latitude position of the density fronts

The density front was defined as the second derivative of the ocean surface density in the latitudinal axis, i.e.,

$$\frac{{\partial }^{2}}{{\partial y}^{2}}\left({\rho }_{surf}\left(x,y,t\right)\right)$$

where ${\rho }_{surf}$ is defined by the linear equation ${\rho }_{surf}=\alpha T+\beta S$. In this case, the values -0.15 and 0.78 were considered for $\alpha$ and $\beta$, respectively.

### NATL effects of precipitation and evaporation induced changes in salinity

The effects of precipitation and evaporation on the salinity in the region of 40° N–60° N and 60° W–20° W (Supplementary Fig. 4b) were obtained via a simple formula that computes the total amount of water that came from precipitation (dilution) and the total amount of water lost due to evaporation (concentration), in respect of the total amount of salt water from a grid cell (horizontally) and the number of cells (n) inside the mixed layer depth (MLD)^54^:

$${\int }_{40N}^{60N}{\int }_{60W}^{20W}\left(S\left(x,y\right) - \frac{P\left(x,y\right)}{MLD\left(x,y\right)}+\frac{E\left(x,y\right)}{MLD\left(x,y\right)}\right)dxdy -{S(\overline{x,y})}$$

in which $S$ is salinity, $P$ is precipitation and $E$ is evaporation. The *MLD* is obtained based on the number of cells inside the mixed layer depth rounded to the closest integer.

### The components of poleward heat and salt balance

According to^37^, the time average of the poleward heat transport is:

$$\overline{HT } = {\int }_{-H}^{0}{\rho }_{0}\,{c}_{p}\,{\left[\overline{\upsilon \theta} \right]}\left({\lambda }_{2} - {\lambda }_{1}\right) cos\,\left(\varphi \right)\,a\,dz$$

where the overbar denotes a time average over a year, brackets are the zonal average at any depth, ${c}_{P}$ is the specific heat capacity, ${\rho }_{0}$ is the sea water density, *v* and $\theta$ are the meridional velocity and the potential temperature of sea water, respectively, H is the depth of the basin, *a* is the earth radius, $\varphi$ is the latitude, and λ_1_ and λ_2_ are the western and eastern longitude boundaries. Reference^37^ decomposes $\overline{\left[\nu \theta \right]}$ into four parcels.

$\left[\overline{\nu }\right] \left[\overline{\theta }\right]$, the “overturning” component.

$\left[{\overline{\nu }}^{*}{\overline{\theta }}^{*}\right]$, the “gyre effect” component.

$\overline{\left[{\nu }{^\prime}{\theta }{^\prime}\right]}$, the “seasonal overturning” component.

$\overline{\left[{\nu }^{*}{^\prime}{\theta }^{*}{^\prime}\right]}$, the “transient eddies” component.

where the asterisk is the deviation from the zonal average. The last two parcels, the “seasonal overturning” and “transient eddies” components, are considered negligible in this study. In an analogous way, the salt poleward transport components are estimated using an equivalent basic equation with the salinity *S*:

$$\overline{HS } = {\int }_{-H}^{0}{\rho }_{0} {\left[\overline{\nu S}\right]}\left({\lambda }_{2} - {\lambda }_{1}\right)\,cos \left(\varphi \right)\,a\,dz$$

### Supplementary Information

Supplementary Figures.

## Data Availability

The data files that support the findings of this study are available from http://ftp.cptec.inpe.br/pesquisa/oceanmc/CMIP5/output/INPE/BESM-OA2-5/.

## References

[1] Rind D (2018). Multicentury instability of the Atlantic meridional circulation in rapid warming simulations with GISS ModelE2. J. Geophys. Res. Atmos..

[2] Gent PR (2018). A commentary on the Atlantic meridional overturning circulation stability in climate models. Ocean Model.

[3] Sévellec F, Fedorov AV, Liu W (2017). Arctic sea-ice decline weakens the Atlantic Meridional Overturning Circulation. Nat. Clim. Change..

[4] Rahmstorf S (2015). Exceptional twentieth-century slowdown in Atlantic Ocean overturning circulation. Nat. Clim. Change..

[5] Yang Q (2016). Recent increases in Arctic freshwater flux affects Labrador Sea convection and Atlantic overturning circulation. Nat. Commun..

[6] Lenton TM (2008). Tipping elements in the Earth’s climate system. Proc. Natl. Acad. Sci..

[7] Caesar L, McCarthy GD, Thornalley DJR, Cahill N, Rahmstorf S (2021). Current Atlantic Meridional Overturning Circulation weakest in last millennium. Nat. Geosci..

[8] Thomas MD, Fedorov AV (2019). Mechanisms and impacts of a partial AMOC recovery under enhanced freshwater forcing. Geophys. Res. Lett..

[9] Bamberg A (2010). Reduced North Atlantic Central Water formation in response to early Holocene ice-sheet melting. Geophys. Res. Lett..

[10] Liu Z (2009). Transient simulation of last deglaciation with a new mechanism for Bølling–Allerød warming. Science (80-)..

[11] Caesar L, Rahmstorf S, Robinson A, Feulner G, Saba V (2018). Observed fingerprint of a weakening Atlantic Ocean overturning circulation. Nature.

[12] Hawkins E (2011). Bistability of the Atlantic overturning circulation in a global climate model and links to ocean freshwater transport. Geophys. Res. Lett..

[13] Stouffer RJ (2006). Investigating the causes of the response of the thermohaline circulation to past and future climate changes. J. Clim..

[14] Rahmstorf S (2005). Thermohaline circulation hysteresis: A model intercomparison. Geophys. Res. Lett..

[15] McManus JF, Francois R, Gherardi J-M, Keigwin LD, Brown-Leger S (2004). Collapse and rapid resumption of Atlantic meridional circulation linked to deglacial climate changes. Nature.

[16] Boers N (2021). Observation-based early-warning signals for a collapse of the Atlantic Meridional Overturning Circulation. Nat. Clim. Change..

[17] Liu W, Liu Z (2013). A diagnostic indicator of the stability of the Atlantic Meridional Overturning Circulation in CCSM3. J. Clim..

[18] McCarthy GD (2015). Measuring the Atlantic Meridional Overturning Circulation at 26°N. Prog. Oceanogr..

[19] Srokosz MA, Bryden HL (2015). Observing the Atlantic Meridional Overturning Circulation yields a decade of inevitable surprises. Science (80-)..

[20] Nobre P (2013). Climate simulation and change in the Brazilian climate model. J. Clim..

[21] Veiga SF (2019). The Brazilian Earth System Model ocean–atmosphere (BESM-OA) version 2.5: Evaluation of its CMIP5 historical simulation. Geosci. Model Dev..

[22] Capistrano VB (2020). Assessing the performance of climate change simulation results from BESM-OA2.5 compared with a CMIP5 model ensemble. Geosci. Model Dev..

[23] Casagrande F (2016). Arctic sea ice: Decadal simulations and future scenarios using BESM-OA. Atmos. Clim. Sci..

[24] Taylor KE, Stouffer RJ, Meehl GA (2012). An overview of CMIP5 and the experiment design. Bull. Am. Meteorol. Soc..

[25] Rahmstorf S (1996). On the freshwater forcing and transport of the Atlantic thermohaline circulation. Clim. Dyn..

[26] Peltier WR, Vettoretti G, Stastna M (2006). Atlantic meridional overturning and climate response to Arctic Ocean freshening. Geophys. Res. Lett..

[27] Wang W, Huang RX (2004). Wind energy input to the Ekman layer. J. Phys. Oceanogr..

[28] Mikolajewicz U, Vizcaíno M, Jungclaus J, Schurgers G (2007). Effect of ice sheet interactions in anthropogenic climate change simulations. Geophys. Res. Lett..

[29] Li H, Fedorov A, Liu W (2021). AMOC stability and diverging response to Arctic sea ice decline in two climate models. J. Clim..

[30] Liu W, Fedorov A (2022). Interaction between Arctic sea ice and the Atlantic meridional overturning circulation in a warming climate. Clim. Dyn..

[31] Liu W, Fedorov A, Sévellec F (2019). The mechanisms of the Atlantic Meridional Overturning Circulation slowdown induced by Arctic Sea ice decline. J. Clim..

[32] Weijer W, Cheng W, Garuba OA, Hu A, Nadiga BT (2020). CMIP6 models predict significant 21st century decline of the Atlantic meridional overturning circulation. Geophys. Res. Lett..

[33] Fröhle J, Handmann PVK, Biastoch A (2022). Major sources of North Atlantic Deep Water in the subpolar North Atlantic from Lagrangian analyses in an eddy-rich ocean model. Ocean Sci..

[34] Liu, W., Fedorov, A. V., Xie, S.-P. & Hu, S. Climate impacts of a weakened Atlantic Meridional Overturning Circulation in a warming climate. *Sci. Adv.* **6**, (2020). 10.1126/sciadv.aaz4876 PMC7319730 32637596

[35] Dixon KW, Delworth TL, Spelman MJ, Stouffer RJ (1999). The influence of transient surface fluxes on North Atlantic overturning in a coupled GCM Climate Change Experiment. Geophys. Res. Lett..

[36] Gregory, J. M. *et al.* A model intercomparison of changes in the Atlantic thermohaline circulation in response to increasing atmospheric CO_2_ concentration. *Geophys. Res. Lett.* **32**, (2005).

[37] Bryan K (1982). Poleward heat transport by the ocean: Observations and models. Annu. Rev. Earth Planet. Sci..

[38] Liu W, Fedorov AV (2019). Global impacts of Arctic Sea Ice loss mediated by the Atlantic Meridional Overturning Circulation. Geophys. Res. Lett..

[39] Cheng L (2020). Improved estimates of changes in upper ocean salinity and the hydrological cycle. J. Clim..

[40] Hu Y, Fu Q (2007). Observed poleward expansion of the Hadley circulation since 1979. Atmos. Chem. Phys..

[41] Lu J, Vecchi GA, Reichler T (2007). Expansion of the Hadley cell under global warming. Geophys. Res. Lett..

[42] Hu Y, Huang H, Zhou C (2018). Widening and weakening of the Hadley circulation under global warming. Sci. Bull..

[43] Lüschow V, Marotzke J, von Storch J-S (2021). Overturning response to a surface wind stress doubling in an eddying and a non-eddying ocean. J. Phys. Oceanogr..

[44] Lohmann K (2021). Response of Northern North Atlantic and Atlantic Meridional Overturning Circulation to reduced and enhanced wind stress forcing. J. Geophys. Res. Ocean..

[45] Griffies, S. M. Elements of MOM4p1. *NOAA/Geophysical Fluid Dynamics Laboratory Ocean Group Tech. Rep. 6* (2009).

[46] Winton M (2000). A reformulated three-layer sea ice model. J. Atmos. Ocean. Technol..

[47] Large WG, Yeager SG (2009). The global climatology of an interannually varying air–sea flux data set. Clim. Dyn..

[48] Giarolla E (2015). Equatorial Atlantic Ocean dynamics in a coupled ocean–atmosphere model simulation. Ocean Dyn..

[49] Nobre P, De Almeida RA, Malagutti M, Giarolla E (2012). Coupled ocean–atmosphere variations over the South Atlantic Ocean. J. Clim..

[50] Figueroa SN (2016). The Brazilian Global Atmospheric Model (BAM): Performance for tropical rainfall forecasting and sensitivity to convective scheme and horizontal resolution. Weather Forecast..

[51] Xue Y, Sellers PJ, Kinter JL, Shukla J (1991). A simplified biosphere model for global climate studies. J. Clim..

[52] Buckley MW, Marshall J (2016). Observations, inferences, and mechanisms of the Atlantic Meridional Overturning Circulation: A review. Rev. Geophys..

[53] Nyadjro ES, Subrahmanyam B, Shriver JF (2011). Seasonal variability of salt transport during the Indian Ocean monsoons. J. Geophys. Res..

[54] Sumner DM, Belaineh G (2005). Evaporation, precipitation, and associated salinity changes at a humid, subtropical estuary. Estuaries.

