# Supplementary material for: AMOC decline and recovery in a warmer climate
Source: Sci Rep. 2023 Sep 23;13:15928. doi: 10.1038/s41598-023-43143-5 (PMC10517999; doi:10.1038/s41598-023-43143-5)
Supplement: Supplementary file 1 — Supplementary Figures. [file 41598_2023_43143_MOESM1_ESM.pdf]

Supplementary Material for
# AMOC decline and recovery in a warmer climate

Paulo Nobre[1*], Sandro F. Veiga[2], Emanuel Giarolla[1], André L. Marquez[1], Manoel B. da Silva Jr.[1], Vinícius B. Capistrano[3], Marta Malagutti[1], Julio P. R. Fernandez[1], Helena C. Soares[1], Marcus J. Bottino[1], Paulo Y. Kubota[1], Silvio N. Figueroa[1], José P. Bonatti[1], Gilvan Sampaio[1], Fernanda Casagrande[1], Mabel C. Costa[1], Carlos A. Nobre[4]

[1]Center for Weather Forecasting and Climate Studies (CPTEC), National Institute for Space Research (INPE), Cachoeira Paulista 12630-000, São Paulo, Brazil

[2]School of Atmospheric Sciences and Key Laboratory of Mesoscale Severe Weather/Ministry of Education, Nanjing University, Nanjing, China

[3]Institute of Physics, Federal University of Mato Grosso do Sul (UFMS), Campo Grande 79070-900, Mato Grosso do Sul, Brazil

[4]Institute for Advanced Studies, University of São Paulo, São Paulo 05508-050, São Paulo, Brazil

Content: Supplementary figures

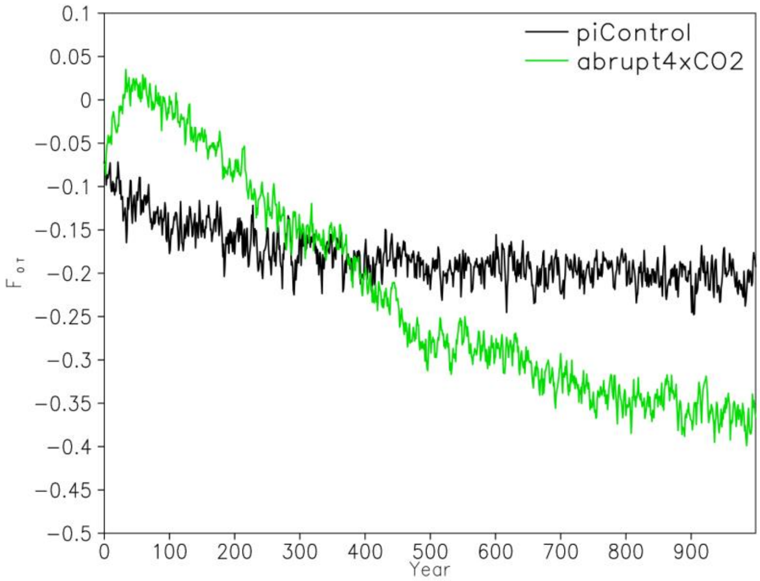


Supplementary Figure 1 – Time series of the overturning component of the freshwater transport ($F_{OT}$)[2,40], at 33° S, for both BESM2.5 piControl and abrupt4xCO2 experiments.

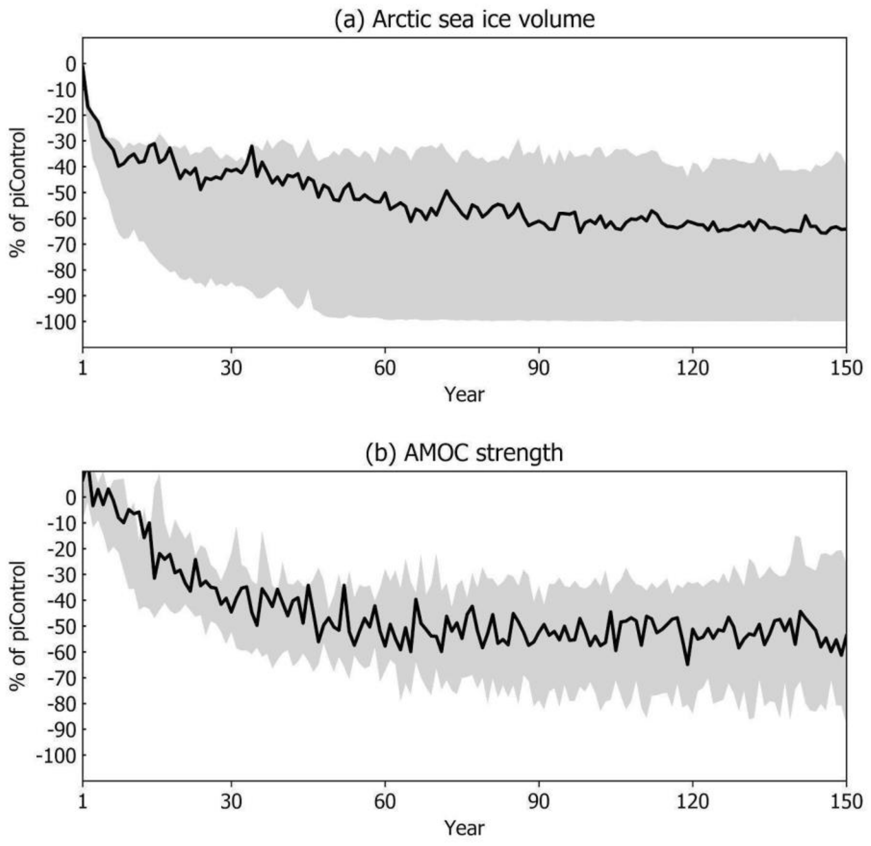


Supplementary Figure 2 - Abrupt4xCO2 minus piControl percent-change time series relative to each model's own piControl run of (a) the Arctic sea ice volume and (b) the AMOC strength using the CMIP5 models. The black line represents the BESM2.5 outcome and the gray shadow is limited by the minima and maxima values given by the following models: BCC-CSM1.1, CanESM2, MIROC-ESM, HadGEM2-ES, MRI-CGCM3, GISS-E2R, CCSM4, GFDL-CM3, GFDL-ESM2G, GFDL-ESM2M. The maximum strength of the AMOC was obtained as an average value in the region defined by a box surrounding the latitudes 25º N–30º N and a depth of 600–1000 m for all models.

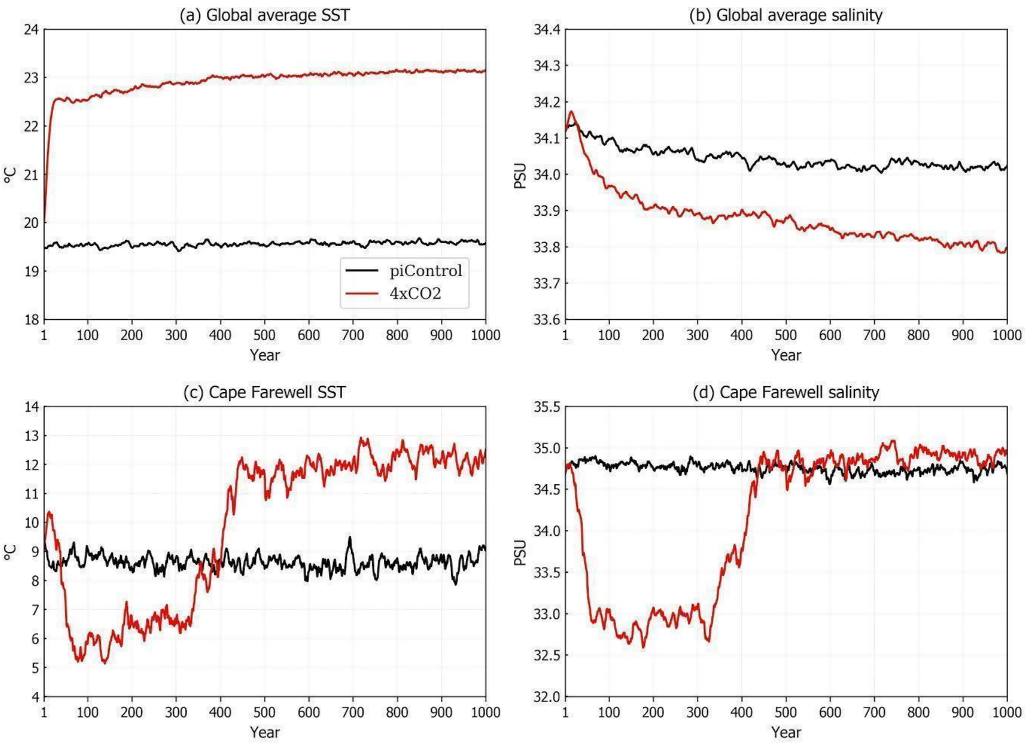


Supplementary Figure 3 - Global-averaged (a) SST (ºC) and (b) sea surface salinity (PSU), and Cape Farewell (59º N, 44º W) (c) SST and (d) SSS with a 10-year running mean-smoothed time series. Abrupt4xCO2 (red) and piControl (black) contours.

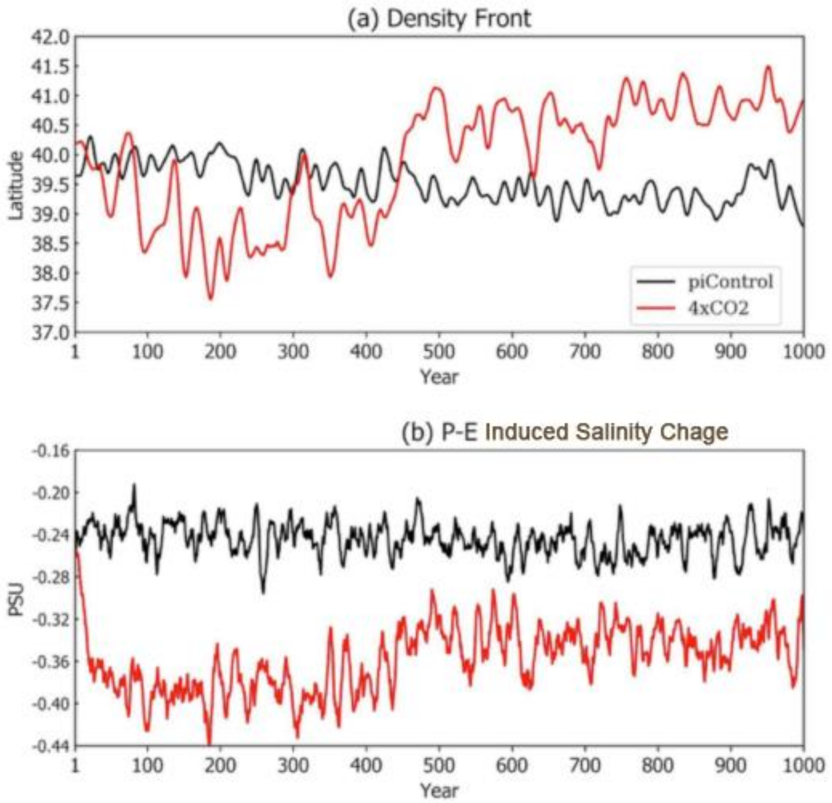


Supplementary Figure 4 - (a) the latitudinal position of the NATL density front and (b) the effects of precipitation minus evaporation on surface salinity over the NATL (40º N–60º N, 60º W–20º W), with a 10-year running mean time series for the Abrupt4xCO2 (red) and piControl (black) lines.

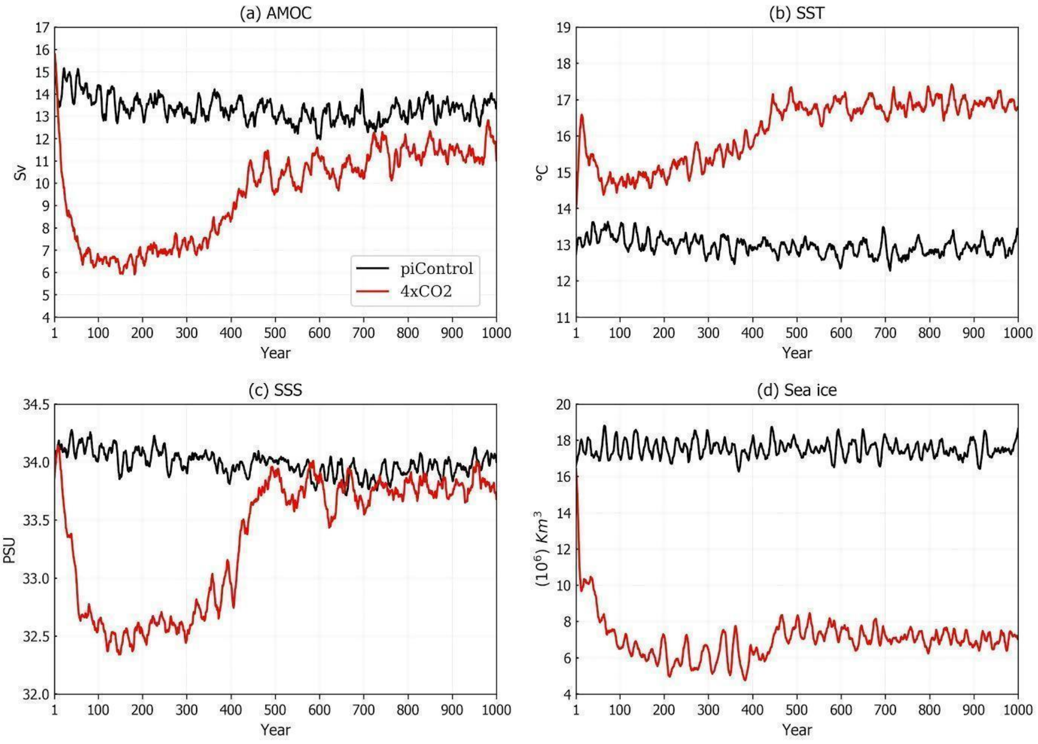


Supplementary Figure 5 - (a) Maximal AMOC strength (computed in a box limited by the latitudes 25º N–30º N at a depth of 600–1000 m), (b) SST (ºC) and (c) SSS (PSU) over the NATL (40º N–60º N, 60º W, 0º W), and (d) Arctic sea-ice volume ($10^6$ $m^3$), 10-year running mean time series for the Abrupt4xCO2 (green) and piControl (black) lines.

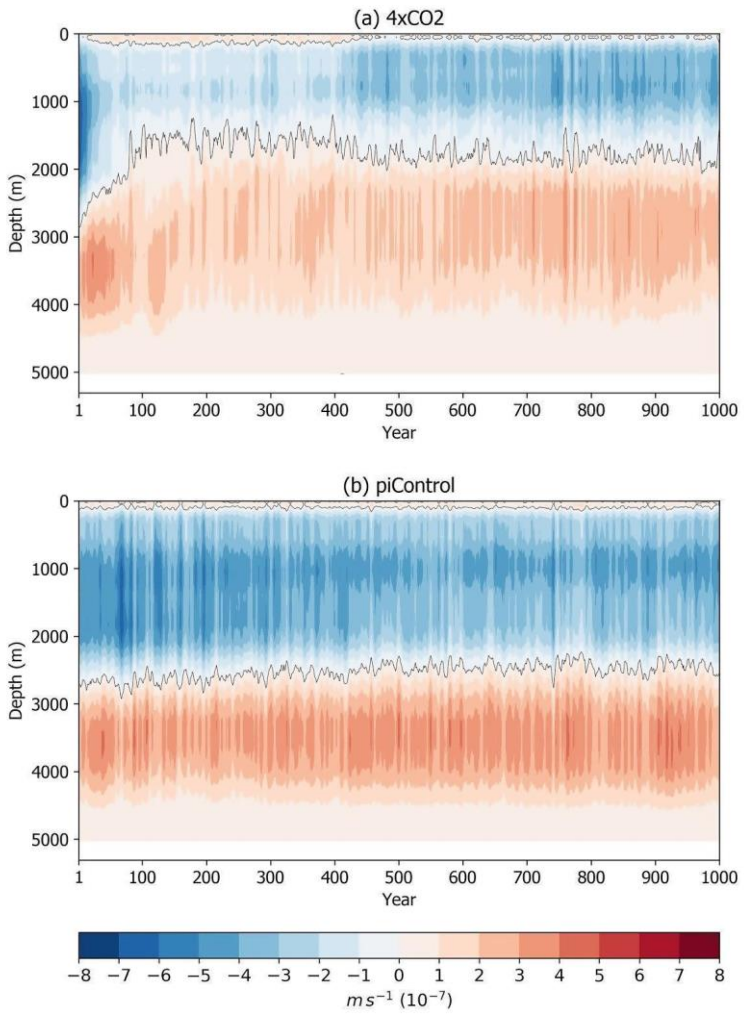


Supplementary Figure 6 - Depth x area averaged (40º N–60º N; 60º W–0º W) vertical velocity (Wt) time series smoothed over a 10-year running mean for (a) the Abrupt4xCO2 and (b) piControl experiments. The black contours indicate the depth level of zero vertical velocity. Positive (negative) values of Wt are upward (downward).

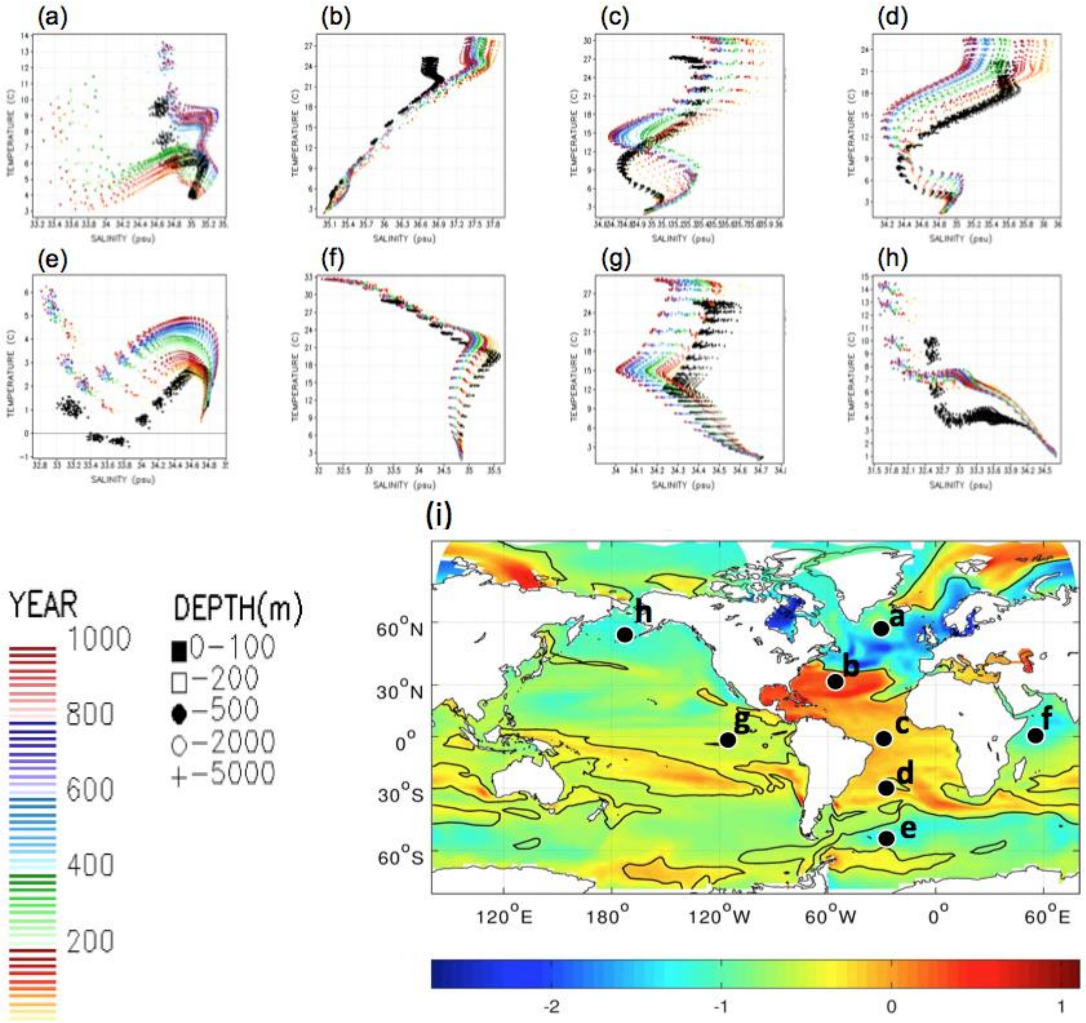


Supplementary Figure 7 - T-S scatter plots for the Abrupt4xCO2 (colors, indicating simulation year) and piControl (black) simulations area averaged in 10º x 10º lon-lat degree, 10-year averages (color scale above) for all ocean depths (depth scale symbols above) at (a) 30º W, 60º N; (b) 60º W, 30º N; (c) 30º W, 0º N; (d) 30º W, 30º S; (e) 30º W, 60º S; (f) 60º E, 0º N; (g) 120º W, 0º N; (h) 180º W, 50º N (depicted by black circles in (i)). The Abrupt4xCO2 minus piControl density anomaly map shown in (i) for simulation years 150–-180 (shades, density units color bar at the base of the panel). The map was generated using MATLAB R2020a (www.mathworks.com) and the plots using GrADS 2.2.0 (http://cola.gmu.edu/grads/).
